# Historical Evolution of the Public Hospital System in Spain, 1963–2019: A Path to Equity in Healthcare Access?

**DOI:** 10.1093/shm/hkaf058

**Published:** 2025-07-20

**Authors:** Margarita Vilar-Rodríguez, Daniel Peñín-Agra, Bruno Casal Rodríguez

**Affiliations:** Department of Economics (Economic History), Facultad de Economía y Empresa, University of A Coruña, 15071 A Coruña, Spain; Department of Economics and Economic History, Facultad de Ciencias Económicas y Empresariales, University of Sevilla, 41018 Sevilla, Spain; Department of Economics (Applied Economics), Facultad de Economía y Empresa, University of A Coruña, 15071 A Coruña, Spain

**Keywords:** healthcare, hospital, inequality, Spain, twentieth to twenty-first centuries

## Abstract

This article contributes to the academic debate on the impact of disparities in the spatial distribution of healthcare resources on the quality of the public health system from a historical perspective. In particular, it analyses the evolution of inequalities in the provincial distribution of public hospital beds in Spain during the Franco dictatorship and the more than four decades of the current democracy. The long study period under consideration and the new statistical data used make it possible to examine to what extent the impact of political, economic and socio-demographic factors affected the equitable distribution of hospital coverage in the country.

Hospitals today have become strategic sites of scientific research, medical training and clinical care, and also the focal points of the Spanish health system and welfare state.^1^ The recent global health emergency brought about by the COVID-19 pandemic has demonstrated this complex strategic role. At the same time, it has encouraged studies on the need to improve the management and geographical distribution of material and human resources in the health sector.^2^ Unequal access to (limited) health resources is a matter of great concern for managers and the population in general, due to its effects on people’s state of health.^3^ The principles for allocating resources in the health sector have been diverse, and have been changing over time in accordance with the allocation mechanisms and policies implemented. These have ranged from utilitarian principles, promoting a distribution of resources based on maximising the population’s health and well-being, to egalitarian principles, which foster a distribution of resources that reduces the existence of inequalities between people.^4^ Simply increasing the resources of a health system does not guarantee a direct improvement in the population’s health outcomes; it is rather how these resources are managed and distributed that determines improvements in overall health.^5^ There is sufficient empirical evidence to affirm that a decisive factor in health inequalities in the population is the existence of inequalities in the historical distribution of health resources.^6^ However, high hospital bed and doctor-population ratios at the national level do not necessarily guarantee adequate resources throughout the country. In this respect, a territorial approach is fundamental to assess equity in the distribution of health resources and to better understand the elements that condition its evolution.

This article is a contribution to the academic debate on the impact of disparities in the spatial distribution of healthcare resources on the quality of the public health system from a long-term perspective. In particular, the literature suggests that a more equitable allocation of health resources increases the overall quality of the health system.^7^ This idea has engendered a proliferation of studies aimed at analysing and understanding different patterns of health resource allocation.^8^ Specifically, the literature dealing with the role of spatial factors may be classified into three types of studies.^9^ One type focuses on the geographical distribution and the characteristics of the health system (measured by the number of clinics, hospitals, doctors, beds, etc.), associated in some cases with population distribution. Another concentrates more on the role of the transport system to take people to these destinations, including in some cases elements of physical geography or socio-cultural factors. A third type considers the characteristics of the people who use the health services (by sex, race and/or socio-economic level). Within this framework, and for the case of Spain, some authors conclude that the regional pattern of health infrastructures and healthcare needs basically responds to differences in past investment and geographical and socio-economic factors, a result that reinforces the importance of a historical perspective.^10^ In the same vein, other authors also add the relevant role of socio-demographic factors in worse access to hospital care. In particular, they highlight the weight of the population over the age of 65, demographic dispersion and the percentage of the population located in rural areas, especially mountainous ones.^11^

Starting from the context outlined above, this study intends to analyse inequalities in the provincial distribution of beds in public hospitals in Spain during the Franco dictatorship (1939–75), the political transition (1976–82) and democracy. This broad period of analysis makes it possible to analyse the different stages of the creation of the public hospital system in Spain. This began with a stage of restricted healthcare and a lack of coordination between hospitals providing compulsory sickness insurance (*Seguro Obligatorio de Enfermedad*; hereinafter SOE). This eventually led to the creation of a modern hospital system within a national health system in a framework of universal and decentralised healthcare. The different contexts and the lower quality of the data available during the dictatorship have led to two study approaches, depending on the period: a more qualitative approach for the dictatorship and the transition and a more qualitative one for the period of democracy. In particular, for this latter period, this article offers an important empirical contribution, including a novel methodology, to the studies available in the Spanish historiography in this field.^12^ In this case, the availability of public hospital beds by province becomes a key variable for understanding the evolution and geographical distribution of healthcare in the country with a focus on equity. With the use of this tool, it will be possible to address four key aspects for the case of Spain. First, how geographical inequality in the distribution of beds has evolved in the country’s public hospital system. Second, the stage in which the foundations of the current geographical distribution in this field were established. Third, the extent to which the devolution of healthcare to the autonomous communities (regions) affected the equity of distribution. Fourth, the extent to which socio-demographic factors, such as an ageing population, population size and density, life expectancy or per capita income, have affected the equality of distribution.

The historiography has studied the historical creation of hospital systems in different countries.^13^ The origins are distant and diverse, but initially a mixed economy of medical care prevailed, with a combination of public and private charity, state action and private payments.^14^ The public hospital systems of Western Europe and Latin America appear to share a historical process based on a combination of philanthropy and tax-based financing, along with the coexistence of different types of mutual sickness insurance related to the substitution of income for primary care in the late nineteenth century. Some countries had already implemented a more complete social health insurance, which included hospital coverage, such as the case of Germany in 1883.^15^

The changes to the hospital system in each country during the twentieth century were subject to their medical, social, political and economic history. However, in the mid-twentieth century, these different hospital developments were transformed into more integrated and regulated systems. Different factors combined to produce an increasingly important role for public hospitals. Increased income and the modernisation of the tax system were indispensable to finance the costs, but other important factors included the implementation of policies aimed at universal coverage and an unprecedented development of biomedicine and associated technology, especially curing methods.^16^ At the end of the twentieth century, hospital access in most European Union countries was by means of a universal and compulsory medical insurance scheme within a broader system of social protection. Let us see what happened in the case of Spain.

## The Public Hospital System in Spain: Keys to Understanding Its Historical Development during the Dictatorship and the Transition to Democracy

Spain was one of the few Western European countries that had not passed a compulsory sickness insurance in the first third of the twentieth century. This delay meant that a public hospital system inherited from the nineteenth century, mainly oriented towards charity, was maintained. Provincial and municipal councils bore the main burden of these deficient infrastructures.^17^ This strategy provided a first hospital map, heavily weighted towards provincial capitals, where most of these hospitals were located. SOE was finally passed in 1942 (launched in 1944) and it became a key element in the dictatorship’s social policy. It was managed by the Ministry of Labour, under the control of the Falange, the single and official party of the Franco dictatorship. The main network of provincial and municipal public hospitals, which were dependent on the Directorate General for Health (*Dirección General de Sanidad*), attached to the Ministry of the Interior (*Ministerio de Gobernación*)—under the influence of another political ‘family’ of the dictatorship—was excluded from the insurance.^18^ In view of the lack of funding and infrastructures, hospital coverage under the SOE was postponed and only surgical emergencies were treated via agreements with private clinics.^19^ Consequently, the public hospital map in Spain was mainly comprised of hospitals with old or obsolete facilities geared more towards providing asylum than curing illnesses.^20^ In many of these, only patients in possession of an official certificate of poverty were admitted, while fees were established for the rest of the population.

SOE adopted the features of a Bismarck-like system, funded by the social contributions of employers and workers in a context of very low wages and with very little state contribution.^21^ Its development was slow, in terms of both affiliation and coverage and provisions. In fact, up to the 1960s, the insurance only applied to low-paid industrial workers in a country where almost half of the active population worked in agriculture. Indeed, until the 1960s, social welfare in rural Spain was more in the nature of a charitable act than a social security system, as was acknowledged by the authorities.^22^ Consequently, in 1963, even less than 40 per cent of the total population was protected, including both insured and beneficiaries (basically family members who lived with insured persons).^23^ Moreover, during these first years, SOE was limited to primary care, access to medicine, hospitalisation——but only for surgery—and small economic benefits.

Within this context, the public hospital system in Spain at the start of the 1960s was a veritable labyrinth with multiple proprietorships (Table 1). Different hospitals were dependent on a number of ministries, with no coordination between them. These were Presidency of the Government (hospitals of the Falange and special hospitals); Ministry of the Interior (charity); Ministry of Labour (*residencias sanitarias*); Ministry of Education (clinics); Ministry of Justice (penitentiary hospitals); and the Ministries of the Army, Navy and Air Force (military hospitals). Apart from this plethora of ministries, there were several other bodies with hospitals, such as the National Anti-Tuberculosis and Chest Disease Trust. During the almost forty years of dictatorship, some changes were made to this classification, although organisational chaos, lack of coordination and duplication of functions continued.

**Table 1.** Public hospitals in Spain by type of proprietorship in 1949 and 1963

	1949		1963	
	Number of establishments	Number of beds	Number of establishments	Number of beds
Military	67	18,245	48	14,367
State (P.N.A. y E.T., DGS^a^, other)	128	18,196	164	26,300
INP (SOE)	36	1,356	56	11,991
SGM	41	2,321	45	2,230
Provincial council	140	37,021	120	37,254
Municipal council	325	11,940	156	8,640
Total public	737	89,079	589	100,782

*Source* 1949: Statistical Yearbook of Spain (*Anuario Estadístico de España*), 1951, p. 684; *Source* 1963: National Hospital Catalogue (*Catalogo Nacional de Hospitales*) updated on 13 June 1966, BOE (official state gazette) of 13 June 1966, 140, 7389-7427. The 1963 Catalogue also included hospital infrastructure in the colonies: Fernando Po (4), Río Muni (11) and Spanish Sahara (5), all under the Presidency of the Government. This source also includes the hospitals of the *Secretaría General del Movimiento* (S.G.M.) and the National Anti-Tuberculosis Trust (*Patronato Nacional Antituberculoso y de las Enfermedades del Tórax*; P.N.A. y E.T.).

^a^DGS = Directorate General for Health (*Dirección General de Sanidad*).

These hospitals functioned in isolation, without common parameters in management, treatment of patients or training of doctors, and they had very disparate characteristics.^24^ Some of these hospitals had a very large capacity and were housed in architecturally obsolete buildings and operated more like charitable asylums than functional hospitals.^25^ These hospitals existed alongside other smaller ones that had proliferated in previous decades as surgical centres in response to the requirements of the SOE. On the other hand, the National Health Facilities Plan (*Plan Nacional de Instalaciones Sanitarias*; hereinafter PNIS) was launched in the 1950s, with the aim of constructing a hospital network for the SOE.^26^ The building of large hospitals, known as *Residencias Sanitarias*, for the SOE progressed very slowly due to a lack of funding, while hospital coverage remained limited. Many of these infrastructures were underused after completion, due to insufficient equipment and a shortage of stable, hierarchical staff and specialists.^27^

In light of these serious shortcomings, the dictatorship passed a law on hospital coordination in 1962, which was intended to create a national hospital network, regardless of proprietorship, open the hospitals to doctors and patients and improve funding. However, this initiative scarcely had any practical impact.^28^ This desolate hospital scenario greatly discredited hospital care among the Spanish population up until the 1960s.^29^Thus, most hospitals in Spain were considered to be obsolete institutions, with poor hygienic conditions, and focussed on treating the poor and people with few resources. In other words, the mentality was quite similar to what could be found in the nineteenth century.^30^ As a result, the hospital attendance rate in Spain in 1967 was 33 admissions per 1,000 inhabitants, well below the figures of most Western European countries (80–120 admissions per 1,000 inhabitants).^31^

Despite all these limitations, the SOE helped create a new hospital-centric culture in Spanish society from two points of view:^32^ it increased demand for healthcare and there was a gradual increase in confidence in the medical attention provided by new diagnostic procedures, medical specialties and hospital care. Rural Spain lagged behind more urban areas in this process of cultural change until, at least, the implementation of a special social security scheme for agricultural workers (*Régimen Especial Agrario de la Seguridad Social*; REASS), established by Law 38/1966 of 31 May. This new legislative framework was intended to provide agricultural workers with a similar degree of social protection as workers in the industrial and service sectors, but the process was not very straightforward in an environment of rural depopulation.^33^ Hence, the projects aimed at bringing new diagnostic tests and therapeutic techniques to rural communities, which had previously received less attention and been restricted to the worst health facilities, were actually abandoned during the 1970s. This was because it had become evident that these small establishments, equipped with a small number of beds, generated unsustainable costs with regard to both staff and technical equipment.^34^

At the end of the 1960s, the prevailing environment allowed for the passage of a law establishing the Social Security (*Ley de Bases de la Seguridad Social*). This law, which was actually implemented in 1966, increased hospital coverage and specialist services, although it did not address universal health coverage. Nor did it resolve the problem of insufficient financing under a nineteenth-century tax system and a Social Security that was dependent on social contributions in one of the countries with the lowest wages in Western Europe.^35^ Nevertheless, in this context, new debates emerged in Spain about the need for hospital reform. Under these circumstances, the need to guarantee access to such a basic service as hospital care for the whole population was being advocated. This would involve a new strategy for the spatial distribution of public hospitals, moving beyond purely economic criteria and propaganda.^36^ As part of this new strategy, hospital planning at this time was guided by reform in two respects. One of these was a process of regionalisation—dividing the country into homogeneous regions and creating a coordinated and tiered hospital network. The other was hierarchical structuring—reorganising the internal clinical services of hospitals. This second element transformed both the distribution of hospital medical services and specialised medical training.^37^ With respect to regionalisation, a decree was promulgated to divide the national territory into eleven hospital regions and to promote plans to establish hospital networks in each region. In most of these regions, this simply led to creating a register of hospitals, but without taking any further measures. There were two exceptions: Catalonia and Asturias. In these two cases, plans for hospital regionalisation were drawn up, with hospitals classified on three levels: regional, intermediate and local hospitals.^38^ These two cases, however, were the exception rather than the rule. In this respect, it is important to bear in mind that in many cases, there may have been little political resolve to promote these changes, but most important of all was the state’s lack of fiscal capacity.

It was not until the start of the transition to democracy that there was a profound reform of the public hospital system in Spain. In this new political stage, agreements were proposed in relation to the need to create a decentralised national health service with universal coverage and funded by taxes.^39^ Two key elements in the development of the public hospital system in Spain were finally secured. First, the advance of the PNIS entailed the construction of a provincial network of *Residencias Sanitarias* throughout the country. Second, the introduction and development of the modern hospital in Spain ushered in a new management model and saw the introduction of new diagnostic and therapeutic technologies.^40^ These changes were consolidated by a change in the financing model, which was only possible after the tax reform of 1977, which enabled progressive funding through taxation, and the recognition of healthcare as a basic right in the 1978 Spanish Constitution, which paved the way to universal health coverage.^41^ Nevertheless, until the law regulating the general state budget (*Ley de Presupuestos Generales del Estado*) of 1989, which introduced a progressive increase in the weight of taxes, most resources were still dependent on the insufficient social contributions of employers and workers.

In the meantime, the Constitution of 1978 laid the foundations for a decentralised health system via the devolution of responsibilities to regional governments. The 1970s and 1980s were the key period for a profound transformation of the hospital system in Spain. This was the case in terms of technology and architecture, and also in regard to the development of a new system of training doctors.^42^

In the 1970s, two key steps were taken in the reorganisation of this complex hospital map.^43^ First in 1974, an independent body was created called ‘Institutional Administration of National Health’, into which a host of health bodies were integrated. These, roughly translated, were the National Anti-Tuberculosis and Chest Disease Trust, the National Psychiatric Care Trust, the Great State General Charity Hospital, the Niño Jesús Hospital, the National Ophthalmic Institute, the National Hospital of Infectious Diseases, the National Oncology Institute, the Trillo National Institute of Leprosy and Leprosarium, the National Centre for Combating Rheumatic Diseases, maternity and paediatric centres, rural hospitals and emergency centres dependent on the Directorate General for Health, the Central Clinic for Rehabilitation and Spanish Institute of Haematology and Haemotherapy and the National Health Centres of Majadahonda. Second, in 1978 the management of the majority of public hospitals was reorganised with the closure of the National Welfare Institute (INP), which had been the main management body of public sickness insurance during the dictatorship, and the restructuring of the Social Security.^44^ This was when INSALUD was established as the main body for the administration and management of health services in Spain. The process of transformation continued with the devolution of healthcare and the creation of different management bodies in the autonomous communities.^45^

## The Public Hospital System in Spain under Democracy

After long years of debate and political disagreements, the General Health Law (*Ley General de Sanidad*, hereinafter LGS) was passed in 1986. Although this law enshrined primary healthcare as one of its basic pillars, the hospital has continued to be the cornerstone of the Spanish health system, thereby consolidating a markedly hospital-centric system. The lesser development and resourcing of primary healthcare appears to be one of the factors that has most influenced hospitalisation rates during the democratic period. Under this legislation, healthcare resources were, as a general rule, publicly owned and managed. However, it also allowed the possibility of reaching agreements with the private health sector and indirect forms of management.^46^ On the other hand, the LGS established an egalitarian system of coverage for the entire country, funded by taxes. It was also compatible with the decentralisation of the responsibility for healthcare to autonomous community level under an umbrella of interregional solidarity, although with a financing framework yet to be defined.

Universal access and the suppression of public charity in the health sphere was not achieved until the passage of the so-called ‘*Decreto de Universalización*’ (Royal Decree 1088/89) in 1989.^47^ This legislation was also a key element in the coordination of the hospital system and accelerated the process of closure and conversion of many public charity hospitals which had begun in the previous decade.^48^ Within this context, many municipal and provincial hospitals with old, out-of-date facilities and obsolete functions were closed. The implementation of this legislation coincided with the devolution of healthcare to the autonomous communities, a process that began in 1981 and was completed in 2002.

The transfer of responsibilities was not easy to manage with such a hospital-centric health system, which was poorly coordinated and comprised of public hospitals of different proprietorship. Moreover, primary care still needed to be modernised and reformed, while the funding model was insufficient and funds were allocated for specific purposes and were non-transferable. In addition, the National Health Institute (INSALUD) continued to be responsible for health in the ten autonomous communities under a common regime (Article 143 of the Spanish Constitution of 1978) and, in theory, set standards. The process of healthcare devolution was not completed in these regions until 2001.^49^ This was when a new regional funding model started to function, based on Law 21/2001, of 27 December, and funds could now be allocated as needed. This law introduced the principles of financial autonomy and joint responsibility or fiscal accountability, principles of the utmost importance in a genuine process of decentralisation.^50^

What were the main innovations introduced by the funding system established in 2001?^51^ First, health financing was no longer allocated for specific ends but rather it was integrated into the total amount of funds assigned to each autonomous community. Consequently, regional governments now had the responsibility for setting the health budget, with the sole condition that spending could not be less than that of the reference year, 1999. Second, a per capita criterion was established for the distribution of funds, weighted by population dispersion and the total land area of the region, while also taking into account insularity (a criterion applicable to the Canary and Balearic Islands). Furthermore, for financing the management of the health services of the Social Security, the law took into consideration both a protected population and the population over the age of 65 in each region, as well as the difficulties arising from the condition of insularity. Finally, regional governments were endowed with a certain degree of fiscal autonomy in order to regulate certain components of the national tax system.^52^ Nonetheless, the bulk of funding was via the direct transfer to regional governments of part of the tax revenue generated in the region but collected by the central tax authority.

Once the decentralisation of healthcare was completed in 2002, the Spanish parliament passed a law on the cohesion and quality of the National Health Service in June 2003.^53^ The main aim of the law was to achieve a balance between decentralisation and coordination. To this end, the law established mechanisms of coordination and cooperation between public health authorities to ensure the population’s right to healthcare and to guarantee equity, quality and participation as common objectives. The law also specified the areas where collaboration between the central state and the autonomous communities was necessary. For these areas, the legislation proposed a common core of action from the National Health Service, but without interfering in the diversity of organisational forms of management and provision of services inherent to a decentralised state.

In the first decades of the twenty-first century, the public hospital system in Spain has been subject to budgetary adjustments and increasing collaboration with the private sector through agreements and new forms of managing provisions and services.^54^ In any case, the recent pandemic has shown how public hospitals provide a basic service for the population’s well-being, and their accessibility and proximity comprise a basic indicator of equity.^55^

## Methods and Data

In the empirical analysis of the unequal distribution of public health resources at the provincial level, the indicator public beds per 1,000 inhabitants in the province has been used as a proxy.^56^ This indicator displays the density of beds in non-asylum public hospitals for each of the fifty Spanish provinces for the period 1986–2019.^57^ When it comes to determining which publicly owned hospitals to count in this study, only acute medicine hospitals, which include general and specialised hospitals, have been taken into consideration. Hospitals which, by their nature, were intended for confinement (asylums, hospices, etc.) rather than providing treatment and cures have been excluded.^58^ Penitentiary hospitals, mental hospitals and geriatric and/or long-stay hospitals intended for patients with chronic pathologies that require a long period of internment have also been excluded.^59^ Despite their function not being registered, beds in military hospitals have been included in the analysis, as they acted as general hospitals for military personnel, their immediate family dependents, and those doing military service. In Spain, the final push in the construction of much of the hospital infrastructure only took place well into the 1980s. In particular, in 1986, military health facilities accounted for 6.2 per cent of all public and private beds available in the hospital system. The total population covered by military health services in Spain in this period was around one million people.^60^ Eliminating the military hospital beds from the analysis would have led to an underestimation of the density of beds, above all in some provinces where the headquarters of the Ministry of Defence’s captaincies-general were located. Generally speaking, these provincial capitals were also where the main military hospitals were located.

**Fig. 1:**
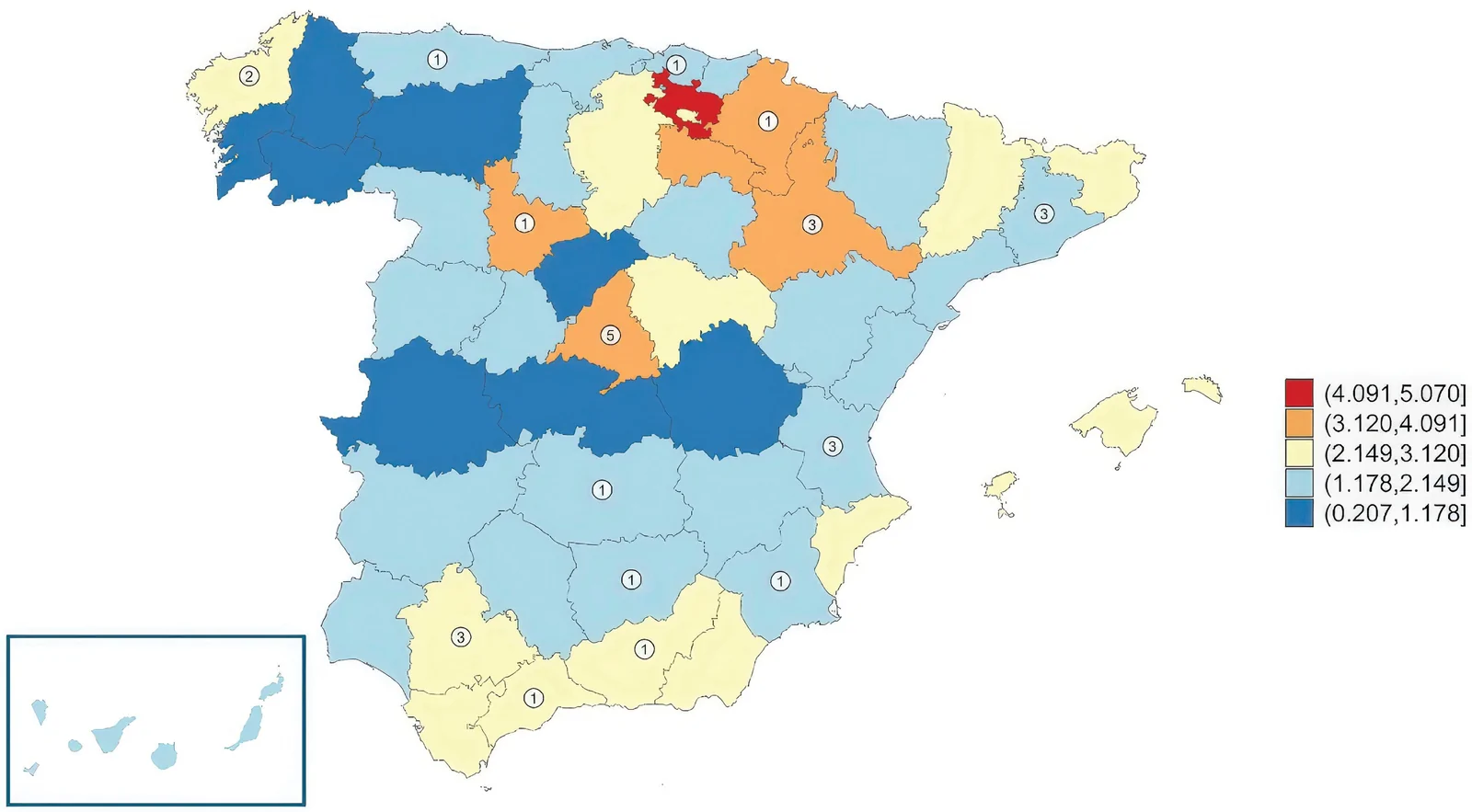
Distribution of public hospital beds at the provincial level in 1963 (per 1,000 inhabitants). *Note*: The numbers included on the map indicate the number of public hospitals with more than 500 beds in each province. *Source*: Prepared by the authors on the basis of the National Hospital Catalogue for 1963. We would like to thank Brais Pociña (University of A Coruña) for his technical assistance in the preparation of this map.

Proprietorship was chosen as the reference for the classification of ‘public’. Our main statistical source comprises the national hospital catalogues. In 1966, the first official catalogue of hospitals and health establishments was published (Figure 1).^61^ This first publication contains errors, but it provides a first reference map of the precarious public hospital system by provinces, described in the first section. The data were updated in 1970, but regular catalogues are not available until the late 1980s. Thus, the first date used for analysis is 1986.^62^ In fact, this year provides an interesting vantage point for observing the hospital map before the implementation of the LGS, the main transfers of healthcare to the autonomous communities and the closure of the public charity hospital network in 1989. Subsequently, 1993 situates us in the second phase of the healthcare decentralisation process, as in these final years of the twentieth century, the transfer of responsibility for healthcare was extended from the autonomous communities considered ‘historical’ to all the other regions (see Table 2).^63^ The year 2005 makes it possible to observe the hospital map once the process of transfer of responsibility for health services to the autonomous communities had been completed.^64^ The next year chosen, 2012, coincides with one of the worst moments of the crisis for the Spanish economy, as it was intervened and forced to apply severe cuts to budget expenditure.^65^ One of the items most affected was health spending. In this respect, a study by the Bank of Spain refers to the period 2009–18 as the lost decade of Spanish healthcare, as while the GDP rose by 8.6 per cent, health expenditure fell by 11.21 per cent.^66^ Some authors point out that this reduction in health spending has, generally speaking, had four main consequences. These are wage cuts, a reduction of staff in the lower professional categories, cheaper medicines and a virtual suspension of investment.^67^ Finally, 2019 shows the provincial public hospital map a few months before the outbreak of the health crisis arising from COVID-19, which also makes it possible to study the geographical distribution of the capacity of public hospital infrastructures to implement contingency measures to deal with the imminent health crisis.

**Table 2.** Density of beds by type of funding for Spanish provinces during the period 1986–2019

	Mean	Median	Standard deviation	Min.	Max.
**Data 1986**					
Total beds per 1,000 inhab.	3.81	3.65	0.99	2.03	6.21
Public beds per 1,000 inhab.	2.89	2.76	0.99	1.04	5.99
Private beds per 1,000 inhab.	0.92	0.89	0.57	0.15	2.32
**Data 1993**					
Total beds per 1,000 inhab.	3.39	3.23	0.64	2.29	5.71
Public beds per 1,000 inhab.	2.72	2.69	0.65	1.17	5.03
Private beds per 1,000 inhab.	0.66	0.53	0.50	0.00	2.13
**Data 2005**					
Total beds per 1,000 inhab.	2.96	2.83	0.55	2.08	4.35
Public beds per 1,000 inhab.	2.40	2.26	0.52	1.37	4.06
Private beds per 1,000 inhab.	0.56	0.45	0.43	0.00	1.60
**Data 2012**					
Total beds per 1,000 inhab.	2.84	2.72	0.54	2.01	4.53
Public beds per 1,000 inhab.	2.34	2.22	0.52	1.20	3.76
Private beds per 1,000 inhab.	0.51	0.40	0.37	0.00	1.63
**Data 2019**					
Total beds per 1,000 inhab.	2.83	2.80	0.55	1.97	4.74
Public beds per 1,000 inhab.	2.33	2.19	0.55	1.27	3.56
Private beds per 1,000 inhab.	0.50	0.46	0.36	0.00	1.58

*Source*: The original data come from the National Hospital Catalogues for 1986, 1993, 2005, 2012 and 2019.

### Measures of inequality

The analysis of the unequal provincial provision of public hospital beds is conducted by calculating and monitoring absolute and relative inequality measures. With regard to the former, the evolution of the standard deviation adjusted for the population is studied. The Gini index, the Atkinson index, the P90/P10 ratio and the coefficient of variation are used as measures of relative inequality in the provision of hospital beds. These measures have been used in other studies that analyse the distribution of health resources in a population.^68^

The Gini index is calculated on the basis of the 50 provinces and shows a range of variation between 0 (all provinces have the same density of hospital beds) and 1 (one province would have all the hospital beds), and is expressed as follows:


$$\begin{array}{l} G=1-\underset{i=0}{\overset{k-1}{\sum }}\;\left(Y_{i+1}+Y_{i}\right)(X_{i+1}-X_{i}). \end{array}$$

where *Y*_*i*_ = the accumulated proportion of the health resource in the province *i*; *X*_*i*_ = the accumulated proportion of population in the province *i*; and *K* = total number of provinces.

The Atkinson index is included in the estimates to take into account aspects of loss of social welfare due to the distribution of the health resource (it uses social welfare functions). The index expresses the proportional difference between the current provision of the health resource and that which would provide the same welfare if the resource was distributed equitably. The same as the Gini index, it shows a range of variation between 0 and 1, increasing with greater inequality in the distribution. In general terms, the Atkinson index may be expressed as follows:


$$\begin{array}{l} A=1-\frac{Y^{*}}{\mu } \end{array}$$

where Y* = the health resource is distributed equally—if the resource was distributed homogeneously it would produce the same welfare as the current distribution; *μ* = current mean provision (*A* = 0 if Y* = μ; A→ *1 as μ* → 0). The index A depends on the specification of the social welfare function and also on individual utility functions. A parameter of society’s aversion to inequality is included in these functions (the greater value of this parameter, the greater society’s opposition to inequality). In this study, values of 0.5, 1 and 2 are used as values of society’s aversion to inequality.

Finally, the P90/P10 ratio measures inequality on the basis of the relationship between the upper bound value of the ninth decile (10 per cent of the provinces with the best provision of the resource) and that of the first decile of the distribution. The coefficient of variation is defined as the standard deviation divided by the mean, and the value is 0 when there is perfect equality in the provision of the resource.

### Determinants of inequality

The analysis of the inequalities in the provincial distribution of public hospital beds was supplemented by an analysis of longitudinal data to help understand the main determinants of this distribution. Population-averaged models were used to estimate the effect of ageing, population density, income and life expectancy on the density of public hospital beds. These models are useful when the number of spatial units and the data available at the time are limited, as is the case here. This analysis combines cross-sectional data on 50 Spanish provinces for 5 years (250 observations). The general model presents the following basic functional form:


$$\begin{array}{l} b_{it}=\alpha +x_{it}\beta +u_{it}, \end{array}$$

in which *i* and *t* represent, respectively, provinces and years; *b*_*it*_ is the beds ratio for the province *i* in the year *t*; x_*it*_ is a vector of time-varying socio-economic covariates; *α* is the intercept and *u*_*it*_ is the error term.

## Results

Table 2 shows the descriptive statistics of the hospital beds per 1,000 inhabitants resource for the five years chosen for the study (1986, 1993, 2005, 2012 and 2019). With regard to public hospital bed provision, the average number remains fairly stable throughout the entire period of study (around 2.5 beds per 1,000 inhabitants). However, a reduction in the provision of public beds can be seen in the table from 1993, which was mainly due to the processes of devolution of health policy to the autonomous communities, which entailed a reorganisation of the public hospital network and an increase in coordination within each region. The dispersion of public hospital beds was reduced from 1993 onwards, falling from a deviation of 1 bed in 1986 to 0.5 beds by the end of the period under study. Without a doubt, the process of devolution of health responsibilities marked the beginning of a process of convergence in the provision of this resource. In more recent years, the reduction in the number of beds in public hospitals was also due to the existence of two supply factors in the regional health systems. One was the implementation of important restrictions to guarantee the fiscal sustainability of the health system due to the financial crisis (Royal Decree-Law 16/2012 redefined the coverage of health services, changing from a universal right to a social security right). The other factor involved changes in the activity of hospitalisation, which led to a reduction in hospital admissions and shorter average hospital stays, basically due to an increase in outpatient surgery, the introduction of early discharge programmes, single-day hospitalisation and the redeployment of acute care resources to chronic care.^69^ The impact of both of these factors on the provision of health resources should be the subject of future analysis, in which it would also be important to take into account demand factors, such as the process of population ageing and the greater prevalence of more complex health conditions (multimorbidity).

Spain has followed the tendency observed in other developed countries, where since 1980 there has also been a general trend of reduction in the number of public hospital beds per inhabitant.^70^ Undoubtedly, the new neoliberal paradigms, which included cuts to welfare spending, and more recently the changes in the activity of hospitalisation, could at least partly explain the trend observed. On the other hand, Spain has remained well below other Western European countries in terms of this ratio (Table 3). Thus, in 2019, France had 3.58 public hospital beds per 1,000 inhabitants; the UK 2.45 and Germany 3.18, compared with the two beds per 1,000 inhabitants in the case of Spain.

**Table 3.** Beds in public hospitals per 1,000 inhabitants during the period 1986–2019

Countries	1986	1993	2005	2012	2019
Austria	7.73	6.57	5.63	5.35	4.99
Denmark	..	..	3.7	..	2.43
Finland	..	..	6.79	5.04	3.21
France	..	..	4.73	3.95	3.58
Germany	..	..	3.68	3.37	3.18
Greece	..	..	3.33	2.97	2.75
Italy	..	..	2.79	2.34	2.11
Norway	..	..	..	3.12	2.63
Portugal	3.16	3.12	2.68	2.45	2.37
Spain	3.03	2.76	2.2	2.06	2.01
UK	..	..	3.72	2.81	2.45

*Source*: Prepared by the authors on the basis of OECD data.

If we observe the historical evolution of the distribution of beds in public hospitals in Spain from a geographical perspective, the following trends can be seen. In the map corresponding to 1963, the provinces with greater resources are the result of a combination of two elements. One is the location in the province of large traditional hospitals belonging to municipal or provincial councils. The other is the location of the regional military hospital (Figure 1).^71^ In 1986 and 1993, after the promulgation of the LGS, some provinces increased their provision of public beds, driven above all by the greater number of large, modern hospitals of the public health insurance network. Meanwhile, as commented above, many old municipal and provincial hospitals with obsolete facilities were closing down. Two important processes were also notable in this period. On the one hand, the case of Madrid. Whereas in 1963, with four beds per 1,000 inhabitants, it occupied second place in the provision of beds, the comparative provision worsened progressively and the ratio of public beds per 1,000 inhabitants has continued well below the average to date (Figure 1). On the other hand, the provinces of Catalonia, where it is easy to observe the region’s public hospital model after the transfer of powers in 1981 (Royal Decree 1517/1981 of 8 July). In this respect, the literature available already highlights as a differentiating element, the fact that 50 per cent of the provision of healthcare in Catalonia was not public at this time. In this context, the recently created *Xarxa Hospitalària d’Utilització Pública de Catalunya* (public hospital network) was made up of public and private hospitals of diverse ownership.^72^ Therefore, in the Catalan case, from the very beginning of the decentralisation of the hospital system, not only were public hospitals integrated into the network, but also private hospitals which, historically, had provided concerted healthcare. In 1993, the Catalan provinces had one public bed less per 1,000 inhabitants than the national average.

**Fig. 2:**
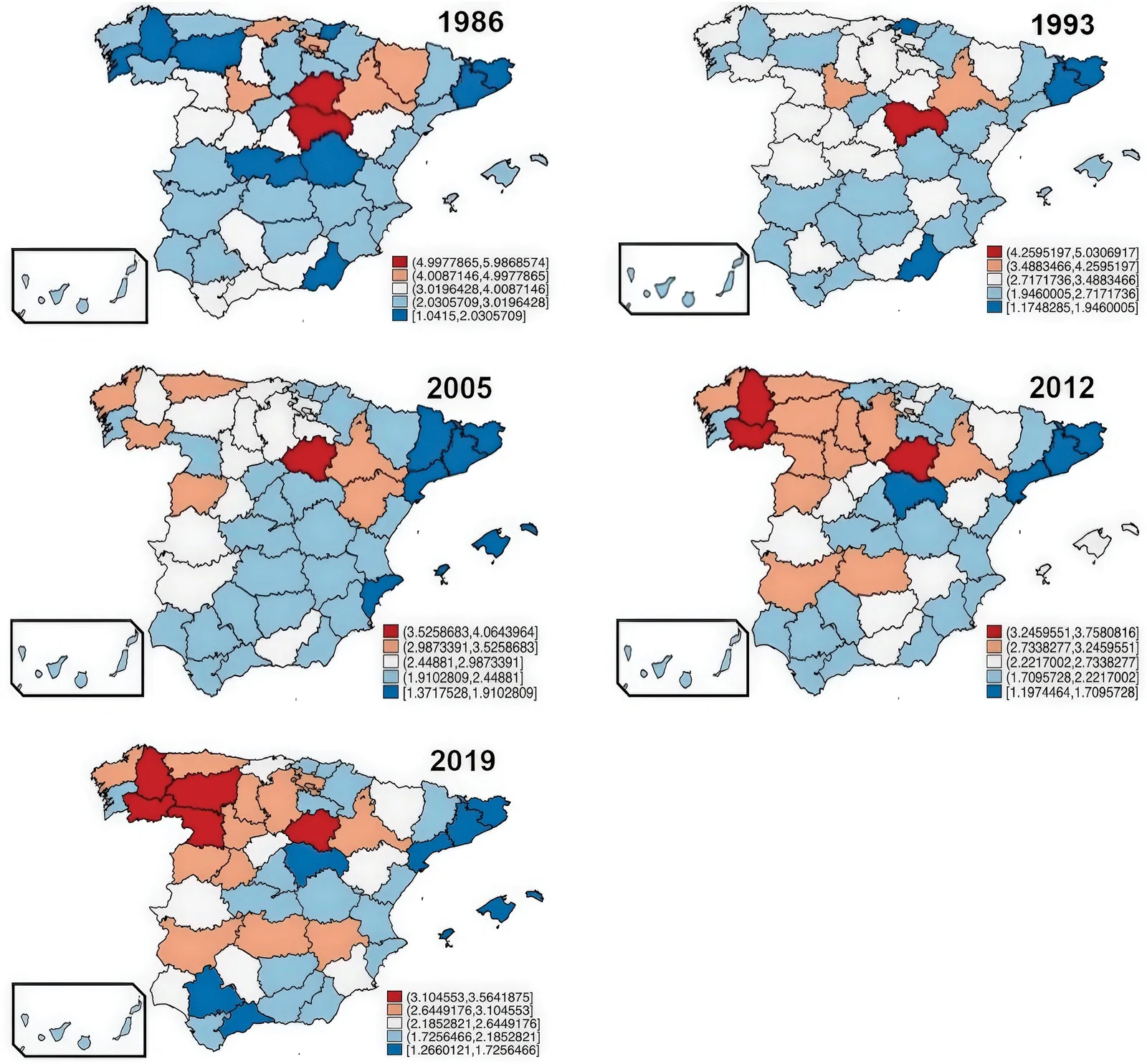
Distribution of public hospital beds at provincial level, 1986–2019 (per 1,000 inhabitants). *Source*: Prepared by the authors on the basis of the National Hospital Catalogues for 1986, 1993, 2005, 2012 and 2019.

At this point it is important to emphasise that in 1993, as well as the reorganisation of the hospital system due to the devolution of healthcare, other demand factors related to the size and ageing of the population were starting to gain importance in the different provisions of public beds that make up the Spanish hospital map (Figure 2).

**Fig. 3:**
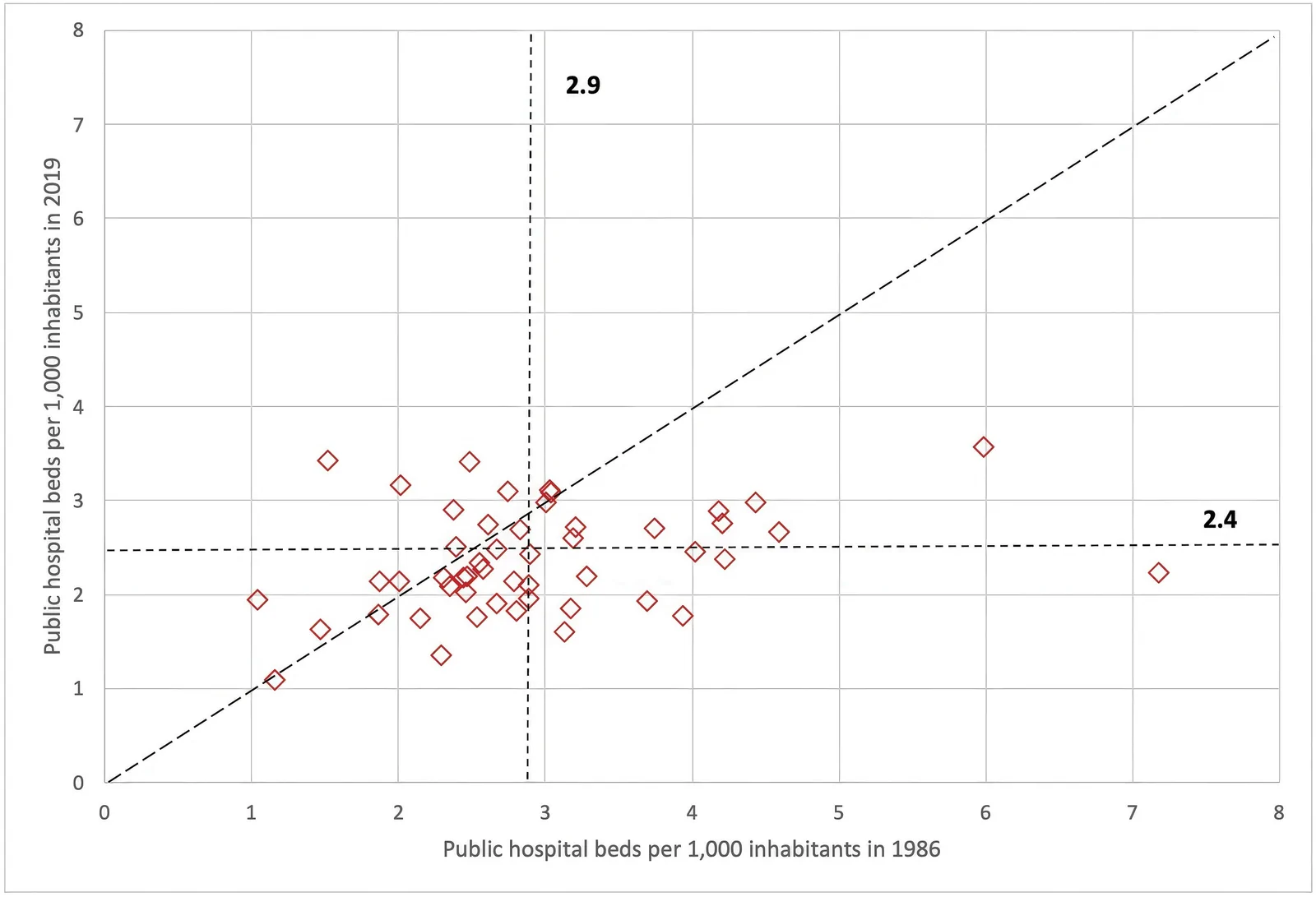
Public hospital beds per 1,000 inhabitants in 1986 and 2019. *Note*: Correlation coefficient = 0.268 (*p* < 0.1). Source: Prepared by the authors on the basis of the National Hospital Catalogues for 1986 and 2019.

In the make-up of the current map of hospital resources, the start of the process of transfer of health services to the autonomous communities has been, without a doubt, the main organisational aspect that explains changes in the allocation of public health resources among regions. The impact and degree of involvement of this process can be analysed by comparing the relationship that exists between the provision of public beds in 1986 and 2019 (Figure 3). Hence, a total of 31 of the 50 provinces have less public hospital beds per inhabitant in 1986 than in 2019 (markers situated below the line of 45°). The majority of provinces are located in the south-west quadrant (21 provinces), indicating that they had below average provision in both years, and a total of 12 provinces had an above-average position in both years (north-east quadrant). These results suggest that the relative position of the majority of provinces on the public bed density indicator has not changed significantly since the start of healthcare transfers to the autonomous communities.

Once the process of healthcare transfers was finished, the maps of the geographical distribution of public hospital beds were clearly conditioned by demographic factors. In general terms, except for the Catalan case explained above, it is the provinces of the so-called ‘empty Spain’ (literally ‘emptied Spain’, ‘España *vaciada*’) that have above-average distribution ratios for public beds.^73^ In this case, the data for 2005 show a negative and significant association between population density and provision of public beds (0.4; *p* < 0.01). It should be taken into account that these provinces with less demographic pressure are also those that have a higher ageing ratio, which leads to a clear paradox: in these areas, less demographic pressure is combined with greater hospital tension due to the greater per capita need for hospital resources. Other healthcare problems that affect these regions of ‘emptied Spain’ should be added to this statement. For example, the deficit of nursing home resources or the modest development of primary healthcare services. In any case, the statistics for 2005 reveal a high association between the ageing index and the provision of hospital beds (0.6; *p* < 0.01).

In fact, the ageing of the population has been playing a fundamental role in the allocation of health resources in recent decades.^74^ It has become one of the main challenges to the sustainability of current health systems, as there is a clear relationship between the increase in health expenditure per capita and the ageing of the population.^75^ The trend of demographic ageing, especially intense in European Union countries, and particularly in Spain, tends to increase public spending on different social provisions, such as pensions, healthcare and social services associated with long-term care.^76^

In Spain, an increase in life expectancy along with a drop in the birth rate brought about a considerable increase in the demographic dependency ratio of the elderly (proportion of people over the age of 64 with respect to the population aged between 16 and 64), which went from 17 per cent in 1975 to 31 per cent in 2022. It also gave rise to a dramatic rise in the ageing index (proportion of the population over the age of 64 with respect to the population under 16 years of age), which rocketed from 35 per cent in 1975 to 133.5 per cent in 2022. In 2014, a case study for the provinces of Catalonia showed the age group with the greatest average health expenditure per person was 80 to 84 years of age (with €2,723 for women and €3,388 for men), followed by the age groups 85 to 89, 70 to 79 and 65 to 69.^77^ These parallel processes suggest the existence of a correlation between the provision of public beds, the ageing of the population and life expectancy.^78^ This is the case at the start of the new millennium, as a result of the ageing of the baby-boom generation (Table 4). Table 4 displays the evolution of the statistic that shows the degree of association between the provision of public hospital beds and the regional ageing indices obtained for the different years of the study. While for the first two years there does not seem to be any type of association between both variables, for later years a significant and increasing relationship can be observed, running in parallel with the demographic changes that have taken place in the Spanish population. In this respect, the regions with higher ageing indices are those that show a greater provision of health resource. The same is observed for the association with life expectancy. Without a doubt, the perspective of necessity lies behind the differences in the provision of public hospital beds in more recent times. However, longitudinal data analysis will be carried out to identify and confirm that population ageing and life expectancy are causes of the distribution of this health resource.

**Table 4.** Statistical evolution of association between ‘public beds per inhabitant’ and ‘ageing index’ and ‘life expectancy at birth’ (1986–2019)

	1986	1993	2005	2012	2019
**Ageing index**	0.1769 (0.2192)	0.1960 (0.1725)	0.5983 (0.0000)	0.7293 (0.0000)	0.7811 (0.0000)
**Life expectancy**	0.4613 (0.0007)	0.3584 (0.0106)	0.2445 (0.0871)	0.2420 (0.0904)	0.2689 (0.0590)

*Note*: *p*-value in parentheses.

*Source*: Prepared by the authors on the basis of the National Hospital Catalogues for 1986, 1993, 2005, 2012 and 2019.

Generally speaking, all the inequality indicators show a reduction of heterogeneity in the distribution of public hospital beds during the period under consideration (Table 5). The greatest changes in the provision of public beds among the fifty Spanish provinces occurred between 1963 and 1986.^79^This is logical since, as mentioned above, between 1975 and 1985 a new type of public hospital was consolidated, where a new form of practising medicine emerged and hospital management was more along the lines of a business organisation, although with specific purposes.^80^ In this process of change towards the so-called modern hospital, many of the old hospitals, with obsolete facilities, were either closed or transformed for other uses. In the meantime, new hospitals were being built that were equipped with new diagnostic and therapeutic technologies.^81^ This process, which coincided with the democratic transition in Spain, laid the foundations for the implementation of the LGS—which among other things included the universality of health coverage—and increased funding for public health. With regard to the reduction in inequality, this can be observed in the years chosen for this study, in which the main transfers of healthcare management to the autonomous communities took place (1993 and 2005).

**Table 5.** Population-adjusted evolution of inequality indicators for the provision of public hospital beds (1986–2019)

	Gini	Atkinson			P90/P10	Coeff. Var.
		*A(0.5)*	*A(1)*	*A(2)*		
**1986**	0.18	0.03	0.06	0.11	2.52	0.34
**1993**	0.13	0.01	0.03	0.06	1.74	0.24
**2005**	0.12	0.01	0.02	0.04	1.64	0.21
**2012**	0.12	0.01	0.02	0.05	1.69	0.22
**2019**	0.13	0.01	0.03	0.05	1.86	0.24

*Source*: Prepared by the authors on the basis of the National Hospital Catalogues for 1986, 1993, 2005, 2012 and 2019.

The chosen indicators also show a slight upturn in inequality from 2012 to 2019. Taking the Atkinson index as an indicator of social welfare linked to the distribution of this health resource, and using the value of 0.5 as a parameter of aversion to inequality, it can be seen that the inequality in the distribution of beds falls until the end of the twentieth century, then it remains constant. For value A (1), the pattern is similar to that observed for the Gini index, whereas for A (2), inequality reaches its minimum a little before the onset of the financial crisis of 2008 and then undergoes a slight rise, which is then maintained over the subsequent years. The same behaviour can be observed with the P90/P10 ratio and the coefficient of variation. After the crisis of 2008, and to meet the objectives agreed with the European Union, the so-called ‘austerity policies’ were implemented in Spain, including in the health sphere, which resulted in cutbacks in funding and temporary closures of some wards, which led to a certain increase in inequality in subsequent years.^82^

Tables 6 and 7 show, respectively, the main descriptive statistics of the variables considered and the results of the longitudinal data analysis on the causes of public hospital bed distribution among Spanish provinces. In line with the correlation analysis, the ageing index presents a statistically significant positive effect on the density of public beds (models 1 and 2). This effect is also confirmed, with a significantly higher estimated impact, when the life expectancy variable is included in the models (models 3 and 4). Including year dummy variables in the models allows us to account for health policy changes, technological advancements or any other time-varying factors that might influence the provision of public hospital beds. The dummy variables for the selected years (year 2019 is omitted) show a positive and significant effect on the density of public beds. Compared to the effect of 2019, the years of the main transfers of healthcare to the autonomous communities (1986 and 1993) show the highest impact on the density of public hospital beds. Although the effect is very small, population density has a negative effect on public bed density.

**Table 6.** Panel data descriptive statistics

Variables by indicator	Obs.	Mean	SD	Min	Max
Public beds per 1,000 inhab.	250	2.535	0.702	1.042	5.987
Population density^a^	250	118.816	154.634	8.684	827.414
Ageing index^b^	250	111.164	54.348	26.08	297.79
999999 per capita (€)^c^	250	18,797.49	5,897.19	7,136.642	37,350.54
Percentage of population	250	0.028	0.035	0.003	0.22
Life expectancy at birth	250	80.117	2.76	74.61	84.93

*Notes*: ^a^Number of inhabitants per square kilometre (data from Instituto Nacional de Estadística, link: www.ine.es); ^b^Persons over 64 years old, for every 100 persons under 16 years of age (data from Instituto Nacional de Estadística, link: www.ine.es); ^c^Adjusted to 2010 prices Leandro Prados de la Escosura, *Spanish Economic Growth, 1850-2023 [dataset]*, Versión 2 (Banco de España (compilador): Repositorio institucional del Banco de España, 2021). Finally, Life expectancy at birth was obtained from the Instituto Nacional de Estadística, link: www.ine.es.

*Source*: Prepared by the authors on the basis of the National Hospital Catalogues for 1986, 1993, 2005, 2012 and 2019.

**Table 7.** Estimated impact of ageing on public provision of hospital beds in Spain (1986–2019)

*Public beds per 1,000 inhab.*	Model (1)	Model (2)	Model (3)	Model (4)
*Ageing index*	0.0032* (0.0004)	0.003* (0.001)		
*Life expectancy*			0.054* (0.019)	0.035*** (0.187)
*Population density*		−0.001* (0.0002)		−0.001* (0.0002)
*Income per capita*		0.21 (0.182)		0.299 (0.217)
*Percentage of population*		1.091 (0.799)		0.286 (0.759)
*Dummy variable for year 1986*	0.461* (0.049)	0.547* (0.148)	0.553* (0.142)	0.605* (0.158)
*Dummy variable for year 1993*	0.358* (0.0419)	0.416* (0.107)	0.454* (0.114)	0.464* (0.118)
*Dummy variable for year 2005*	0.082* (0.0319)	0.086* (0.024)	0.197* (0.061)	0.153* (0.056)
*Dummy variable for year 2012*	0.052* (0.0318)	0.073* (0.028)	0.066* (0.023)	0.083* (0.03)
*R*-squared	within = 0.344 between = 0.236 overall = 0.276	within = 0.366 between = 0.269 overall = 0.3	within = 0.21 between = 0.127 overall = 0.154	within = 0.287 between = 0.142 overall = 0.187
*N*	250	250	250	250

*Notes*: (1) The Hausman test was applied, and the fixed effects model was rejected (Prob < chibar2 = 0.000); (2) Robust standard errors in parentheses; (3) *p < 0.01; **p < 0.05; ***p < 0.1; (4) Public beds per 1,000 inhab. and income per capita in logarithms.

*Source*: Prepared by the authors on the basis of the National Hospital Catalogues for 1986, 1993, 2005, 2012 and 2019. See note in Table 6.

## Conclusions

This article has made it possible to incorporate the case of Spain into the academic debate that has been ongoing in recent decades on the importance of good management and geographical distribution of material and human resources in the health sector and social welfare of a country. In particular, this study analyses the Spanish case by using changes in the geographical distribution of public hospital beds as a key indicator from a long-term perspective. The results have made it possible to verify that the public hospital map in Spain has undergone a profound transformation since 1963. Thus, although it can be seen that the average provision of the country as a whole has remained stable during the period under study, at around 2.5 public beds per 1,000 inhabitants, its distribution has changed radically. The data consolidate the thesis put forward in the historiography that the lack of coordination of the public hospital system in Spain persisted until the 1970s. Moreover, it remained dependent on numerous institutions and was characterised by the coexistence of public charity hospitals with obsolete facilities and a nineteenth-century tradition with new hospitals built under the Franco dictatorship’s PNIS, but underused and with insufficient material and human resources. The coexistence of public hospitals of diverse proprietorship in some cities led to an increased provision of beds, which created a false impression of better health coverage, but also led to greater geographical inequality. During this period, it seems that political interests took priority over the population’s needs and the characteristics of each province in the provision and geographical distribution of public hospital beds.

The end of the dictatorship and the start of the transition to democracy allowed better coordination between public institutions and the beginning of profound change in the management and provision of hospital resources. This new process of creation of a modern public hospital system led to a new concept of open hospital, characterised by profound organisational and management changes. During this process, the first LGS was passed in 1986, which established a National Public Health System with universal coverage, where hospitals comprised the central axis of care, expenditure and training of professionals, while the traditional system of public charity inherited from the nineteenth century was legally eliminated in 1989.^83^ All of this in a context where the process of devolution of healthcare to the autonomous communities had already begun.

All these changes brought about a radical transformation of the public hospital system, which led to greater hospital coordination, better management of the hospitals and greater equity in the geographical distribution of these resources. The new funding framework passed after the transfer of healthcare to the autonomous communities had been completed also helped reduce geographical inequalities, by taking into account differential factors such as the population served, demographic dispersion, the rate of population ageing and insularity. As a result of this new approach, aimed at covering the needs of the most dependent people, equity has improved, along with greater heterogeneity in the provision of public hospital beds. This heterogeneity is evident, for example, in the areas of the so-called ‘empty Spain’, characterised by a high ageing ratio and less demographic pressure, with above-average distribution ratios for public beds. This heterogeneity may also be a direct result of the fact that these areas tend to have a more elderly population and so are less attractive to private hospital groups, where the number of potential customers would not offset the costs of establishing themselves there.^84^ This study has shown that, as a result of this process, and once the modern public hospital system had been consolidated, the correlation coefficients that measure the association between the number of public beds and the ageing rate are significant from 1993 to 2005, unlike the previous periods. The longitudinal data analysis shows that population ageing and life expectancy have affected the distribution of this health resource among Spanish provinces since the beginning of the transfer of health services to the Autonomous Communities. The results also confirm the impact of health policy changes in 1986 and 1993 on the density of public hospital beds.

Finally, the Great Recession of 2008 brought about a change of trend in this process. In particular, inequality indicators rose between 2005 and 2009, and the growing trend continued until at least 2019, the situation prior to the outbreak of the pandemic. During this stage, the results were conditioned by the so-called ‘austerity’ policies imposed on Spain by the European Union, measures that played a fundamental role in the increase in inequality in the provision of health resources.^85^ The measures adopted led to the closure of hospital wards (and a decrease in the number of beds), cuts to services and a reduction in health workers, all accompanied by processes of privatisation in both management and services provided.^86^ The impact of these measures was not homogeneous throughout Spanish territory, which contributed to the increase in inequality between regions.^87^ Nevertheless, the political management of different regional governments as another factor in these differences should not be overlooked.

## Footnotes

1 See Josep Barceló-Prats, Elisa Alegre and Josep M. Comelles, ‘Del hospital de pobres a la cultura hospitalo-céntrica. Economía política y cambio cultural en el sistema hospitalario catalán’, *Kamchatka. Revista de análisis cultural*, 2017, 10, 57–85; Josep Barceló-Prats, ‘Genealogía de la reforma hospitalaria en España’, *Dynamis: Acta Hispanica ad Medicinae Scientiarumque Historiam Illustrandam*, 2021, 41, 27–51; Paloma Fernández-Pérez, *The Emergence of Modern Hospital Management and Organization in the World 1880s–1930s* (Bingley: Emerald Publishing Limited, 2021).

2 See Julen Izagirre-Olaizola, Goizalde Hernando-Saratxaga and María-Soledad Aguirre-García, ‘Integration of Healthcare in the Basque Country during COVID-19: the Importance of an Integrated Care Management Approach in Times of Emergency’, *Primary Health Care Research & Development*, 2021, 22, 1–11.

3 For further information on the key factors related to healthcare inequalities, see Mel Bartley, *Health Inequality: An Introduction to Concepts, Theories and Methods* (Cambridge: Polity Press, 2016). For a greater understanding of the effects of these factors on the equitative distribution of health resources, see, for example, Tuvia Horev, Irena Pesis-Katz and Dana B. Mukamel, ‘Trends in Geographic Disparities in Allocation of Healthcare Resources in the US’, *Health Policy*, 2004, 68, 223–32; Miao Du *et al*., ‘Evaluating the Inequality of Medical Resource Allocation Based on Spatial and Non-Spatial Accessibility: A Case Study of Wenzhou, China’, *Sustainabilit*y 2022, 14, 8331.

4 See Anthony J. Culyer, ‘Equity: Some Theory and Its Policy Implications’, *Journal of Medical Ethics*, 2001, 27, 275–283.

5 For further details, see Derek Yach and David Harrison, ‘Inequalities in Health: Determinants and Status in South Africa’, in K. van der Velden *et al*., eds, *Health matters: Public Health in North-South Perspective* (Amsterdam: Bohn Stafleu Van Loghum, 1995).

6 See Diane McIntyre, *Health Financing. Learning from Experience: Health Care Financing in Low and Middle-Income Countries* (Geneve: Global Forum for the Research Health, 2007), 55; Xiang Yan *et al*., ‘Divergent Distributions of Physicians and Healthcare Beds in China: Changing Patterns, Driving Forces, and Policy Implications’, *Applied Geography* 2022, 138, 102626.

7 To discover more about how the equitable allocation of health resources increases the quality of the health system, see Ronald M. Andersen, Thomas H. Rice and Gerald F. Kominski, eds., *Key Issues in Health Services Policy and Management* (San Francisco: John Wiley &Sons, 2007); Horev, Pesis-Katz and Mukamel, ‘Trends in Geographic Disparities’. For more on the effects of this equitable allocation on economic efficiency, see Christopher J. L. Murray and Julio Frenk, ‘Un marco para evaluar el desempeño de los sistemas de salud’, *Boletín de la Organización Mundial de la Salud: la revista internacional de salud pública*, 2000 3, 149-162, link: https://iris.who.int/handle/10665/58011.

8 For further information on the different patterns of health resource allocation, see Isabel Correia and Paula Veiga, ‘Geographic Distribution of Physicians in Portugal’, *European Journal of Health Economics*, 2010, 11, 383–93; Shinichi Tanihara *et al*., ‘Urbanization and Physician Maldistribution: A Longitudinal Study in Japan’*, BMC Health Services Research*, 2011, 11, 260. https://bmchealthservres.biomedcentral.com/articles/10.1186/1472-6963-11-260.

9 See Gary Higgs, ‘Literature Review of the Use of GIS-Based Measures of Access to Health Care Services’, *Health Services and Outcomes Research Methodology*, 2004, 5, 119–39.

10 See, for example, Jaume Puig Junoy and Ángel López Nicolás, ‘Assessing Health Care Infrastructure at the Regional Level: A Statistical Approach’, *Applied Economics Letters*, 1995, 2, 463–66.

11 For further information, see Francisco J. Goerlich, Joaquín Maudos and Silvia Mollá, *Distribución de la población y accesibilidad a los servicios en España* (Madrid: Fundación Areces, 2021), 81.

12 There are very few studies of the historical creation of the Spanish hospital system from the regional point of view over such a long period as that addressed in this study, except some specific cases, but none of these provide an empirical analysis. For further information on the process of regionalisation of healthcare in Spain during the dictatorship and the transition to democracy, see, for example, Josep Barceló-Prats, Josep M. Comelles and Enrique Perdiguero-Gil, ‘Las bases ideológicas y prácticas del proceso de regionalización de la sanidad en España (1955-1978)’, in M. Isabel Porras, Lourdes Mariño and M. Victoria Caballero, coords., *Salud, enfermedad y medicina en el franquismo* (Madrid: Los Libros de la Catarata, 2019), 146–67. For further details on the process of creation of the modern hospital in Spain before 1986, see the excellent work of Josep Barcelo-Prats, ‘The Emergence of Modern Hospital Planning and Administration in Spain (1966–1986)’, in P. Fernández-Pérez, ed, *Business History of Hospitals in the 20th Century* (Cham: Springer Nature Switzerland, 2024), 83–96. For the period of democracy, see, for example, Gregori Galofré-Vilar and María Gómez-León, ‘Provincial Health Inequalities in Spain Since 1860’, *Research in Economic History*, 2025, 38, 1–25. These authors use annual mortality rates at the provincial level for men and women to estimate changes in regional health inequalities in Spain since 1860. See also Pilar García-Gómez and Ángel López Nicolás, ‘The Evolution of Socio-Economic Health Inequalities in Spain: 1987–2001’, *Electronic Journal*, 2004, DOI: 10.2139/ssrn.563845. In this study, the authors use the probabilities of visiting a doctor, using emergency services and being hospitalised as variables of analysis.

13 See John Henderson Peregrine Horden and Alessandro Pastore, *The Impact of Hospitals 300–2000* (Bern: Peter Lang, 2007); Gorsky, Vilar-Rodríguez and Pons-Pons, *The Political Economy* (Huddersfield: Huddersfield Press, 2020).

14 For further information on how the mixed economy of medical care was created in different countries, see Bernard Harris and Peter Bridgen, eds, *Charity and Mutual Aid in Europe and North America since 1800* (New York: Routledge, 2007) and John Murray, *Origins of American Health Insurance. A History of Industrial Sickness Funds* (New Haven-London: Yale University Press, 2007), for a study of the American case; Bernard Harris, *The Origins of the British Welfare State: Social Welfare in England and Wales, 1800–1945* (Hampshire: Palgrave Macmillan, 2004); and Michael Dreyfus, *Les assurances socials en Europe* (Rennes: Presses Universitaires de Rennes, 2009), for a study of the European case; and Marcel Van der Linden, ed, *Social Security Mutualism: The Comparative History of Mutual Benefit Societies* (Bern: Peter Lang, 1996); Brian J. Glenn, ‘Understanding Mutual Benefit Societies 1860–1960’, *Journal of Health Politics, Policy and Law*, 2001, 26, 638–51, for a study of mutuals.

15 For further information, see Alfonso Herranz, ‘La difusión internacional de los seguros sociales antes de 1945’, in Jerònia Pons and Javier Silvestre, eds, *Los orígenes del estado de bienestar en España, 1900–1945* (Zaragoza: Prensas Universitarias de Zaragoza, 2010), 51–84.

16 To discover more about these factors and their influence on hospital hegemony, see the introduction to Gorsky, Vilar-Rodríguez and Pons-Pons, *The Political Economy*. In particular, an analysis of the increasingly important role of hospitals in different countries, from an internal management focus, can be found in Fernández-Pérez, *The Emergence of Modern Hospital*.

17 For further information on how the provincial and municipal councils assumed the management of these hospitals, and the challenges that these new public managers faced, see Pedro Carasa, *El sistema hospitalario español en el siglo XIX. De la asistencia benéfica al modelo sanitario actual* (Valladolid: Universidad de Valladolid, 1985). For more on the conditioning factors of this process, see Margarita Vilar-Rodríguez and Jerònia Pons Pons, Un siglo de hospitales entre lo público y lo privado (1886–1986) *[Financiación, gestión y construcción del sistema hospitalario español]* (Madrid: Marcial Pons, 2018); Margarita Vilar-Rodríguez and Jerònia Pons-Pons, ‘El papel de hospitales municipales y provinciales en España desde una perspectiva histórica’, *Dynamis: Acta Hispanica ad Medicinae Scientiarumque Historiam Illustrandam*, 2021, 41, 79–110. For Galicia, see Margarita Vilar-Rodríguez, ‘Saúde e atención sanitaria’, in Ramon Villares, coord., Os tempos son chegados. *Un balance da Galicia autonómica, 1981–2021* (Santiago de Compostela: Parlamento de Galicia, 2022).

18 For further details of the internal conflicts within the dictatorship, see Alberto Pieltain, *Los hospitales de Franco. La versión autóctona de una arquitectura moderna* (Madrid: Universidad Politécnica de Madrid, 2003); Jerònia Pons-Pons and Margarita Vilar-Rodríguez, *El seguro de salud privado y público en España. Su análisis en perspectiva histórica* (Zaragoza: Prensas Universitarias de Zaragoza, 2014); Margarita Vilar-Rodríguez and Jerònia Pons-Pons, ‘Competition and Collaboration between Public and Private Sectors: The Historical Construction of the Spanish Hospital System (1942–1986)’, *The Economic History Review*, 2019, 72, 1127–636.

19 For further information on the lack of financial resources during the first stages of the SOE, see Francisco Comín, *Historia de la cooperación entre las cajas: la Confederación Española de Cajas de Ahorros (1928–2007)* (Madrid: Alianza, 2008); Pons-Pons and Silvestre, eds, *Los orígenes del estado del bienestar*.

20 For further details of the deficiencies of the SOE, see Carlos Barciela, ed, *Autarquía y mercado negro. El fracaso económico del primer franquismo, 1939–1959* (Barcelona: Crítica, 2003); Vilar-Rodríguez and Pons-Pons, *Un siglo de hospitales*, 179; Vilar-Rodríguez and Pons-Pons, ‘Competition and Collaboration’.

21 Pons-Pons and Vilar-Rodríguez, *El seguro de salud privado y público*, 245.

22 Boletín Oficial del Estado (Official State Gazette) of 2 June 1966, number 131, p. 6906. For further information, see also Josep Barceló Prats and Daniel Lanero Táboas, ‘From Abandonment to Hospitalisation: Evolution of Hospital Care in Rural Spain (1939–1975)’, *Social History of Medicine*, 2022, 35, 661–81. https://doi.org/10.1093/shm/hkab126

23 E. Martín López, *Estudio socioloógico sobre el seguro de enfemedad en España,* I (Madrid: Ministerio de Trabajo, Gabinete de Sociología, Secretaría General Técnica, Archivo INGESA, manuscrito, 1963). Pons-Pons and Vilar-Rodríguez, *El seguro de salud privado y público*, 127.

24 Barcelo-Prats, *The Emergence of Modern Hospital*, 83–96, 85.

25 Vilar-Rodríguez and Pons-Pons, ‘El papel de hospitales municipales’.

26 For more on the National Health Facilities Plan (PNIS), see Alberto Pieltain, *El hospital. Doscientos años de proyectos* (Madrid: Instituto Nacional de la Salud, 2000); Pieltain, Los hospitales de Franco; Pons-Pons and Vilar-Rodríguez, *El seguro de salud privado y público*, 137.

27 For further details of the deficiencies of this plan, see Vilar-Rodríguez and Pons-Pons, *Un siglo de hospitals*, 180.

28 J. Artigas, *La ley de hospitales como ley de coordinación. Estudios sobre hospitales y Beneficencia*, 1967, 25, 43–46. For further information on the *Ley de Coordinación Hospitalaria* of 1962, see Pieltain, *Los hospitales de Franco*; Pons-Pons and Vilar-Rodríguez, *El seguro de salud privado y público*, 137.

29 Barcelo-Prats, *The Emergence of Modern Hospital*, 83–96.

30 Vilar-Rodríguez and Pons-Pons, ‘El papel de hospitales municipales’.

31 Barcelo-Prats, *The Emergence of Modern Hospital*, 83–96.

32 In line with the arguments of Josep M. Comelles *et al*., ‘Por caminos y veredas. La práctica médica rural bajo el franquismo (1939–1979)’, in José Martínez-Pérez and Enrique Perdiguero-Gil, coord., *Genealogías de la reforma sanitaria en España* (2010), 63–124.

33 Margarita Vilar-Rodríguez and Jerònia Pons-Pons, ‘La cobertura social de los trabajadores en el campo español durante la dictadura franquista’, *Historia Agrar*ia, 2015, 66, 177–210.

34 Daniel Lanero-Táboas, ‘Historia de una infraestructura menguante: la crisis terminal del hospital rural al final del franquismo (1960–1975)’, *Dynamis: Acta Hispanica ad Medicinae Scientiarumque Historiam Illustrandam*, 2020, 41, 111–33. Barceló-Prats and Lanero-Táboas, ‘From Abandonment to Hospitalisation’, 661–81.

35 See more in Margarita Vilar-Rodríguez and Jerònia Pons-Pons, ‘La Ley de Bases de la Seguridad de 1963: ¿una oportunidad perdida?’, in Damián A. González and Manuel Ortiz, eds, *El Estado de Bienestar entre el franquismo y la transición* (Madrid: Sílex, 2020), 125–56.

36 Barceló-Prats, *The Emergence of Modern Hospital*, 83–96.

37 Further information on this process of hierarchical organisation can be found in Barceló, ‘Genealogía de la reforma hospitalaria’, 27–51. For more details on the changes to the training of doctors, see Miguel A. Asenjo-Sebastián, ‘Difícil camino, espléndida realidad: el sistema MIR’, *FEM. Revista de la Fundación Educación Médica*, 2018, 21, 2, 75–78; and Carlos Soler-Durall, ‘Reflexiones sobre el hospital: su función en la formación de médicos’, *Educ Med*, 2010, 13, 71–77.

38 For Catalonia, see Josep Barceló-Prats and Josep M. Comelles, *L´evolució del dispositiu hospitalari a Catalunya (1849-1980)* (Barcelona: PAHCS, 2020); for Asturias, see José García-González, *La implantación del hospital moderno en España. El Hospital General de Asturias, una referencia imprescindible* (Oviedo: Nobel, 2011); Barceló-Prats, ‘Genealogía de la reforma hospitalaria’, 27–51.

39 Vilar-Rodríguez and Pons-Pons, *Un siglo de hospitales*, 237–264; Enrique Perdiguero-Gil and Josep M. Comelles, ‘The Defence of Health. The Debates on Health Reform in 1970s Spain’, *Dynamis: Acta Hispanica ad Medicinae Scientiarumque Historiam Illustrandam*, 2019, 39, 45–72.

40 For further details of the creation of the ‘modern hospital’, see José Luis Temes, ‘Desarrollo del Sistema hospitalario’, *Tribuna Médica*, 1989, 1219, 39–42. García-González, *La implantación del hospital moderno*; and Wenceslao López Albó, *Planeamiento y organización de un hospital moderno* (Salamanca: Biblioteca Marquesa de Pelayo, 2012).

41 See more in Vilar-Rodríguez and Pons-Pons, *Un siglo de hospitales*, 237–64.

42 Barceló-Prats, *The Emergence of Modern Hospital*, 83–96, 85.

43 Decree-law 13/1972, of 29 December, modifying the institutional administration of the Directorate General for Health, dependent on the Ministry of the Interior, and entrusting the restructuring of this management body to the Government. Boletín Oficial del Estado (BOE) of 10 January 1973, 9, 488–90.

44 Royal Decree-law 36/1978, of 16 November, regulating the institutional management of the Social Security, health and employment. BOE 18 November 1978, Núm. 276, 26246–26249.

45 For further information on the process of devolution of responsibility for healthcare and the managing bodies that emerged, see Marqués, *El Sistema Nacional*; Juan Ventura, *Organización y gestión de la atención sanitaria* (Oviedo: Universidad de Oviedo, 2003).

46 Art 90 of the LGS; BOE of 29 April 1986, 12, 15219.

47 BOE of 9 September 1989, 16, 28657–28658.

48 For further information, see Vilar-Rodríguez and Pons-Pons, ‘El papel de hospitales municipales’, 110.

49 For further details of the devolution of healthcare, see Víctor Conde Rodelgo, ‘Los últimos 20 años de los Centros Sanitarios en España’, *Arbor*, 2001, 170, 247–75; Francisco Marqués, *El Sistema Nacional de Salud después de las transferencias sanitarias* (Murcia: Consejería de Sanidad, 2003).

50 BOE of 31 December 2001, 313, 50383–50419. For this part, see Pons-Pons and Vilar-Rodríguez, *El seguro de salud privado y público*, 292; Guillem López i Casasnovas and Ana Rico, ‘La descentralización, ¿parte del problema sanitario o de su solución?’, *Gaceta sanitaria: Órgano oficial de la Sociedad Española de Salud Pública y Administración Sanitaria*, 2003, 17, 319–26; David Cantarero, ‘El traspaso de competencias sanitarias en España’, *Revista de Administración Sanitaria siglo XXI*, 2003, 1, 65–80; Guillem López i Casasnovas, ‘La descentralización sanitaria, la cohesión social y el buen funcionamiento del sistema de salud’, *Mediterráneo económico*, 2016, 30, 401–26.

51 For further details, see BOE of 31 December 2001, 313, 50383–50419.

52 For further details, see BOE of 31 December 2001, 313, 50383–50419.

53 BOE of 29 May 2003, 128, 20567–20589.

54 For further information, see Marciano Sánchez Bayle, *Privatización sanitaria. Análisis y alternativas* (Barcelona: El Viejo Topo, 2019).

55 For further details, see M. Carmen Rodríguez-Blas, *Estadística de gasto sanitario público 2018: Principales resultados* (Madrid: Ministerio de Sanidad, 2021).

56 For further information, see Horev, Pesis-Katz and Mukamel, ‘Trends in Geographic Disparities’.

57 There are considered to be fifty provinces, excluding the autonomous cities of Ceuta and Melilla.

58 For the same reason, centres for the prevention of infectious children’s diseases and hospitals intended to care for sufferers of tuberculosis and leprosy, geriatric and psychiatric patients and those undergoing rehabilitation have also been excluded from the sample for analysis. Moreover, hospitals of a national or international nature have also been excluded, as it is considered that they would create bias as they tend to treat patients from outside the province. Finally, industrial accident and occupational illness mutuals have also been excluded due to considering their main function to be related to rehabilitation.

59 For further details, see Goerlich, Maudos and Mollá, *Distribución de la población*, 77.

60 For further information, see Pablo Gutiérrez-González, ‘Los hospitales militares y la sanidad militar. La transición de un modelo segregado a la creación del ISFAS’, in Vilar-Rodríguez and Pons-Pons, *Un siglo de hospitales*, 367–400.

61 National Hospital Catalogue (*Catalogo Nacional de Hospitales*) updated on 13 June 1966. This was to be revised every year and was intended to provide a basis for a new and more efficient hospital regionalisation. The data were updated in 1970, but regular catalogues are not available until the 1980s. For further information, see García-González*, La implantación del hospital moderno.*

62 National Hospital Catalogue updated on 1 January 1986.

63 National Hospital Catalogue updated to 31 December 1992.

64 National Hospital Catalogue updated to 31 December 2004.

65 To find out more about the effects of the austerity policies, see the National Hospital Catalogue updated to 31 December 2011.

66 Mario Alloza, Júlia Brunet, Victor Forte-Campos, Enrique Moral-Benito and Javier J. Pérez (2022), El gasto público en España desde una perspectiva Europea, Banco de España, Documentos Ocasionales. 2217. Link: https://www.bde.es/wbe/es/publicaciones/analisis-economico-investigacion/documentos-ocasionales/gasto-publico-espana-desde-perspectiva-europea.html (accessed in November 2023).

67 For further information, see Eduardo Bandrés and Rosa González, ‘La reducción del gasto sanitario en España durante la crisis’, *Cuadernos de Información Económica*, 2015, 248, 37–48.

68 For further information, see Hugh Gravelle and Matthew Sutton, ‘Inequality in the Geographical Distribution of General Practitioners in England and Wales 1974–1995’, *Journal of Health Services Research & Policy*, 2001, 6, 6–13; Satar Rezaei *et al*., ‘Relative Inequalities in Geographic Distribution of Health Care Resources in Kermanshah Province, Islamic Republic of Iran’, *EMHJ-Eastern Mediterranean Health Journal*, 2016, 22, 20–27.

69 For further information, see Guadalupe Carmona López *et al*., ‘Impacto de la Crisis Económica en la Salud y en el Sistema Sanitario de España’, *Documento de Trabajo de la Escuela Andaluza de Salud Pública*, 2015.

70 For further information, see Fernández-Pérez, *The Emergence of Modern Hospital*, 5–6.

71 The doctoral thesis defended by Helios Pardel in 1973 indicates that in Spain in the 1960s, the provinces with greater hospital resources are also those that have a higher per capita income. We are aware of this thesis from secondary sources but we have not been able to access it for a direct consultation. Nonetheless, in our analysis for the period of democracy, the relationship between income and the provision of public beds appears to be negative (see Table 7). This could be due to the fact that wealthier regions resort more to private healthcare. Helios Pardell (1973) demonstrates that this statement is also valid, with nuances, today and in more recent decades. See: Helios Pardell, ‘Estudio de la dotación hospitalaria española’ (Doctoral thesis, Universidad Autonomous University of Barcelona, 1973).

72 For further information, see Conde Rodelgo, *Los últimos 20 años*, 261.

73 The term ‘*España vaciada*’ refers to those (above all inland and rural) areas of Spain that have been losing population since the mid-twentieth century and continue to do so. Some authors who have analysed this issue show that access to public services has played an essential role, particularly when the construction of the welfare state made the population feel that there was a penalty for residing in settlements where there were problems to access them. See, for example, María Isabel Ayuda, Pablo Gómez and Vicente Pinilla, ‘Which Rural Settlements Have Lost the Most Population? An Analysis of a Case Study of North-East Spain (Aragón) (1900–2001)’, *Rural History*, 2023, 1–19.

74 For further information on the phenomenon of population ageing and its effects on the allocation of resources, see David Casado Marín, ‘Los efectos del envejecimiento demográfico sobre el gasto sanitario: mitos y realidades’, *Gaceta Sanitaria*, 2001, 15, 154–163.

75 The Organisation for Economic Co-operation and Development (OECD) and the European Union’s Working Group on Ageing Populations and Sustainability establish four main factors in the evolution of health expenditure. The variation in the percentage of the population covered, the increase in the number of people covered by public health will have a direct effect on the increase in health expenditure; the variations in the evolution of real benefits, that is, whether there is an increase in the quantity and quality of services provided; price increases and demographic changes (where population ageing must be emphasised). For further information, see Ángela Blanco Moreno, Rosa Urbanos Garrido and Israel J. Thuissard Vasallo, ‘Distribución del gasto sanitario público por edad y sexo en España análisis de la década 1998–2008’, *Fundación de las Cajas de Ahorros*, 2012, 673.

76 For a greater understanding of the need for greater health expenditure with age, see Casado Marín, ‘Los efectos del envejecimiento demográfico’. For the Spanish case, see José Barea and Enrique Fuentes Quintana, ‘El déficit público de la democracia española’, *Papeles de Economía Española*, 1996, 68, 86–191.

77 For further information, see Emili Vela *et al*., ‘Análisis poblacional del gasto en servicios sanitarios en Cataluña (España): ¿qué y quién consume más recursos?’, *Gaceta Sanitaria*, 2019, 33, 24–31.

78 Although the analysis concentrates on these variables, there are other elements that may also be influencing this behaviour, such as the increase in chronic diseases or the expansion and specialisation of primary care, particularly outpatient services, which now cater to patients who previously necessitated hospitalisation. This aspect has been addressed in various studies, including Win Van Lerberghe, *The World Health Report 2008: Primary Health Care: Now More Than Ever* (World Health Organisation, 2008). The evolution of medical treatments has also led to a reduction in the duration of hospitalisations, implying that a greater number of patients can be accommodated with the same or fewer beds. For further information, see D. Golinelli *et al*., ‘20 Years of Declining Hospital Length-of-Stay in Different European Healthcare Models and Systems’, *European Journal of Public Health*, 2023, 33, ckad160-610. These aspects could be incorporated into future analyses.

79 This statement has been made even though the authors are aware that the 1963 catalogue contained less reliable data than the catalogues published in the 1980s. These results verify what has already been noted from another analysis perspective in Vilar-Rodríguez and Pons-Pons, *Un siglo de hospitales*, 263.

80 As highlighted by Barceló, ‘Genealogía de la reforma hospitalaria’, 51; the new concept of public hospital based on the ‘hospital-company’ model entailed a change of paradigm which involved moving from what could be termed ‘scientific developmentalism’ to the management of human and material resources.

81 Along the lines indicated by García González, *La implantación del hospital moderno*, who points to the Asturias General Hospital as the pioneering centre of this process.

82 In November 2008, the European Commission published a recovery plan, which set out the guidelines for Member States to follow in order to counteract the effects of the financial crisis (EUR-Lex). This European plan served as a basis for the Spanish Plan to Boost the Economy and Employment. The implementation of the Spanish plan led to budgetary adjustments in the health system in the form of cuts to public healthcare. For further information, see Bandrés and González, ‘La reducción del gasto sanitario’, 37–48.

83 Royal Decree 1088/1989, of 8 September, by which Social Security healthcare cover is extended to persons without sufficient economic resources. BOE of 9 September 1989, 216, 28657–28658.

84 For further information, see Margarita Vilar-Rodríguez and Jerònia Pons-Pons, ‘The Penetration of Financial Capital and the Growth of Private Hospital Groups in Europe: The Case of Spain (1975–2022)’, *Medical History*, 2023, 66, 339–59.

85 See Terje Andreas Eikemo *et al*., ‘Desigualdades sociales en salud y sus determinantes: principales resultados de la séptima edición de la Encuesta Social Europea’, *European Social Survey*, 2016, 6. The impact of the Great Recession and the effect of austerity policies applied to healthcare go beyond the scope of this study. For further details in this respect, see, for example, Jeroen Spijker, ‘Los efectos de la Gran Recesión y las políticas de austeridad en la salud de la población española’, *Gac Sanit*, 2020, 34, 220–22.

86 See Kees Gool and Mark Pearson, ‘Health, Austerity and Economic Crisis: Assessing the Short-Term Impact in the OECD Countries’, *OECD Health Working Papers*, 2014, 76.

87 A similar conclusion was reached from a different study perspective in Amaia Bacigalupea *et al*., ‘Austeridad y privatización sanitaria en época de crisis: ¿existen diferencias entre las comunidades autónomas?’, *Gaceta Sanitaria*, 2016, 30, 47–51.

